# Home infusion with Elosulfase alpha (Vimizim^R^) in a UK Paediatric setting

**DOI:** 10.1016/j.ymgmr.2017.10.012

**Published:** 2017-11-05

**Authors:** Niamh Finnigan, Jane Roberts, Jean Mercer, Simon A. Jones

**Affiliations:** Willink Biochemical Genetics Unit, Manchester Centre for Genomic Medicine, Central Manchester University Hospitals NHS Foundation Trust, Saint Mary's Hospital, UK

**Keywords:** Homecare, Morquio syndrome, MPS IVA, Vimizim

## Abstract

Enzyme replacement therapy is the only available treatment for Mucopolysaccharidosis type IVA (MPS IVA, Morquio syndrome). The treatment is lengthy and invasive involving weekly intravenous infusions of 4–5 h. This can cause significant disruption to normal family life so the provision of a safe and effective homecare service is essential. In order to deliver a safe service, robust standards must be in place; this includes appropriately trained members of homecare staff, detailed management for infusion related reactions (IRR) and appropriate venous access. In this report we demonstrate the criteria required to ensure a successful home treatment programme and describe our experience thus far.

## 1 Introduction

Mucopolysaccharidosis type IVA (MPS IVA, Morquio A Syndrome) is an autosomal recessive lysosomal storage disease (LSD) caused by a deficiency in the enzyme *N*-acetylgalactosamine-6-sulfatase (GALNS) due to a mutation in the GALNS gene located on chromosome 16q24.3 [1]. Infants with MPSIVA usually appear normal at birth, however, due to the accumulation of storage material in tissues and organs, leading to cellular dysfunction they progressively develop profound skeletal and joint abnormalities alongside a range of non-skeletal manifestations [2], [3], [4]. Such manifestations can include impaired respiratory function, valvular heart disease, obstructive sleep apnoea, hearing impairment, corneal clouding, spinal cord compression and dental abnormalities [5].

Morquio A syndrome can often be distinguished from other types of MPS disorders by a typical short trunk dwarfism with a short neck; skeletal manifestations tend to be more extensive than in other MPS disorders. Alongside this, hypermobility of distal joints is a significant feature and is characteristic of Morquio A syndrome [6]; Morquio A syndrome has not been associated with cognitive impairment [7].

The most common gene mutation is present in < 9% of Morquio patients giving rise to a wide heterogeneity with regard to clinical presentation, severity of disease and rate of progression [8]. Some patients may present with a more classical phenotype associated with short stature and severe skeletal and joint abnormalities, whereas some patients do not have a characteristic presentation but may show atypical signs such as hip stiffness and pain. Attenuated patients tend to present later in life, are taller and have less spinal disease. Due to the heterogeneous and progressive nature of the disease, the management of patients is often challenging and requires a multidisciplinary approach [6].

In April 2014, Elosulfase alpha (Vimizim) was licensed in the EU and funded as a treatment for MPS IVA in the UK by NICE (National Institute for Health and Care Excellence) in December 2015. It is delivered on a weekly basis via an intravenous infusion over an average of four to five hours. Findings from the clinical trials involving a total of 235 patients with Morquio syndrome showed that Vimizim significantly improved endurance, decreased urinary KS levels and was generally well tolerated [9]. Of the 235 patients enrolled in the clinical trial, 16 (6.8%) experienced signs and symptoms consistent with anaphylaxis. The timing of these reactions were often as early as 30 min after the beginning of the infusion to 3 h after the completion of the infusion. All but two were able to receive further infusions of Elosulfase alpha with infusion rate adjustment and/or medical intervention [10], [11]. Based on the outcome of the phase 1 and 2 study, a multicenter, double blind, placebo controlled phase 3 study was performed to assess the efficacy and safety of infusions with Elosulfase alfa 2.0 mg/kg every week and every other week [12]. The study showed significant improvement in endurance of 22.5 m in 6MWT distance during 24 weeks of treatment with Elosulfase alfa at 2.0/mg/kg/week as compared with placebo. No significant impact was observed with alternate weekly dosing [6].Vimizim is shown to provide positive and meaningful changes in several clinical parameters. Treatment should be commenced as soon as possible after diagnosis is made; however results of treatment may be variable due to the significant heterogeneity of the condition [5].

Although ERT has been proven to be effective, there are many factors that impact upon both the patient and their families. It is invasive, and the prolonged infusion time often requires time off school, work or college. The families often have to travel a significant distance to a dedicated treatment centre such as our own which can often cause a financial burden. Many patients may find frequents visit stressful and time consuming whilst hospital visits may reduce feelings of frustration and isolation as they are often given the opportunity to meet other families and patients suffering with the same condition [13], [14]. By transferring into homecare perceived benefits may include less disruption to activities of daily living, less disruption to normal family life and more involvement in their own care leading to greater independence [14]. Home therapy allows to alleviate the long term burden of a lifelong therapy and has been shown to significantly improve compliance by effectively avoiding missed infusions due to non-clinical reasons and increasing scheduling flexibility [20], [21]. Long term compliance is vital in order to maintain the efficacy of enzyme replacement therapy as suggested in long term studies for other forms of MPS disorders [17], [18], [19] therefore the importance of delivering treatment is a convenient setting for the patient and family is vital.

**Fig. 1:**
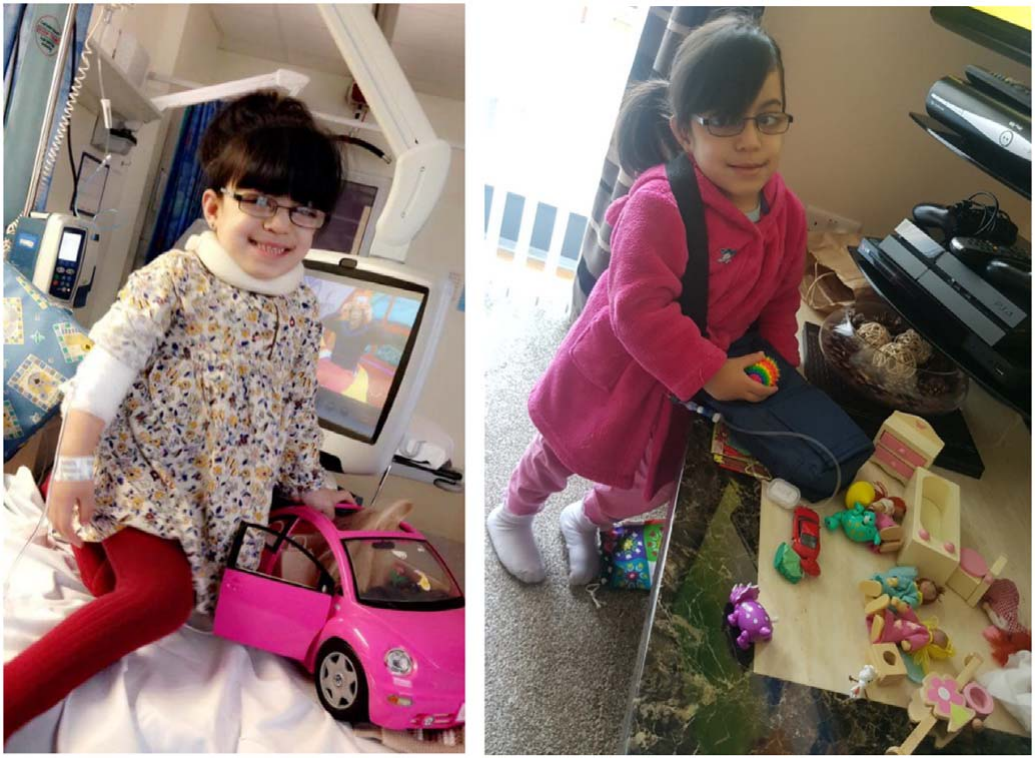
Patient receiving Vimizim in the hospital and homecare setting.

There are also risks associated with home therapy including the possibility of infusion related reactions (IRR) when the patient is not in a hospital environment, this may vary from minor pyrexia or a rash to a full anaphylactoid reaction. Therefore, it is vital that patients are selected for homecare in order to maximize the benefits and minimize the risks. Nevertheless, despite these Fig. 1 risks patients with LSD's still prefer home therapy [13], [14], [15]. In this publication we review our experience with home enzyme replacement therapy treatment in children with MPS IVA and demonstrate the criteria required to ensure a successful home treatment programme.

## 2 Patients and methods

Patient demographics, clinical features, infusion related reactions (IRR) and the use of pre-medications are indicated in Table 1, Table 2. The details of Vimizim dosing are given in Table 3.

**Table 1:** Patient demographics.

Number of MPS IVA	23
Number treated with Vimizim in centre	20 (14 currently on homecare) 3 untreated 6 treated at other centre's
Age range	4–16 years
Average age at diagnosis	46.6 months (3.88 years)
Severe: Attenuated	18:5 (12:2 currently on homecare)
Number on nocturnal respiratory support	30%

**Table 2:** Homecare data from the 14 patients.

Total number of LSD patients (all children) receiving any homecare ERT at the center	71
IRRs in hospital	6 (42.8% of current patients)
Use of pre-medications	14 (100%)^a^
Duration of ERT in hospital prior to home treatment (weeks)	10–15 weeks^b^
Duration of ERT at home in weeks (at time of reporting)	18–200 (mean–109)
Number now having school based infusions	6
Number of current patients using TIVAD's for treatment	10 (71.4%)
Number of patients using weekly cannulation for treatment	4 (28.6%)

a As mandated from clinical trial protocols.

b These figures are based on treatment naive patients only as those on clinical trials remained in hospital for a longer period before transition to home infusions.

**Table 3:** Infusion details for Elosulfase alpha (Vimizim) [6].

Vimizim		
Dose	2 mg/kg weekly	
Dilution	< 25 kg: dilute in 100 ml of 0.9% saline > 25 kg: dilute in 250 ml 0.9% saline	
Rate of administration	In 100 ml: Start rate 3 ml/h After 15 min, 6 ml/h After 15 min, 12 ml/h After 15 min, 18 ml/h After 15 min, 24 ml/h After 15 min, 30 ml,hr. After 15 min, 36mlhr	In 250 ml: Start rate 6 ml/h After 15 min, 12 ml/h After 15 min, 24 ml/h After 15 min, 36 ml/h After 15 min, 48 ml/h After 15 min, 60 ml,hr. After 15 min, 72mlhr
Total duration of infusion	4–5 h	

## 3 Criteria followed for the safe transfer of patients to home therapy

The below criteria demonstrate the criteria used to select the patient cohort described in Table 1, Table 2 for a safe transfer to home therapy.

## 4 Patient

### 4.1 Fully established on Elosulfase alpha

Patients need to be stable on Elosulfase alpha with either no IRR's or IRR's that have been managed appropriately. Some patients may never experience an IRR. However, if this does occur reactions need to be managed in an appropriate manner with adjustment in their pre-medication regime and/or infusion rate changes in order to stabilize the patient in a hospital setting prior to transfer. The minimum duration of before transition to the home setting should be 12 weeks.

### 4.2 Established intravenous access

The patient should have good peripheral venous access or a totally implanted venous access device (TIVAD) in situ. The family should also be aware of how to care for such devices and the risks that are associated with these. Such risks may include line infections, swelling, redness and to avoid close contact sports that may knock or damage the TIVAD. Within the hospital setting there is also a robust service provided by the play therapy team, who are vital when assisting younger children who are needle phobic.

### 4.3 Each patient assessed individually

It is very important that all patients are considered for homecare on an individual basis. The patient is reviewed prior to homecare by the LSD team. Some patients may have further complications to consider such as severe airway disease that may complicate the effect of IRRs and require for them to stay in hospital for a longer period of time. Others may have behavioral issues or severe needle phobia that needs addressing.

## 5 Home healthcare team

### 5.1 A member of the healthcare at home team should meet the patient prior to transfer

This is important to ensure that all the patients' needs and expectations are met. This will often include the member of staff coming to the treatment centre to introduce themselves to the patient and family and to observe venous access and the pre-medication regime. The homecare team also carries out a home visit to ensure there is an appropriate environment to prepare and administer Elosulfase alpha.

The family doctor should be made aware of the patient transfer to home infusions and the presence of any IRRs or of a TIVAD.

### 5.2 Homecare staff should be experienced

The nurses should receive the appropriate training in relation to administering enzyme replacement therapy and caring for children with lysosomal disorders. Nurses will also undergo compulsory resuscitation and anaphylaxis training on an annual basis. Each nurse will carry an individualised kit for any IRRs that may occur.

### 5.3 Homecare team must provide a weekly report

The homecare team must submit a report to the centre on a weekly basis which documents any reactions that may have occurred or difficulties with accessing the patients.

### 5.4 Regular contact with the hospital

There should also be a direct phone line to the LSD centre. Homecare staff should inform the prescribing team of any issues they may be having or to obtain any advice. This may include if the patient has been unwell recently to ensure it is still safe to proceed with the infusion.

The healthcare company should provide a dedicated 24-h nurse on-call service via the patient's local regional nursing team, and a dedicated customer service team Monday to Friday 08:00–18:00 [10].

Once all of the above criteria have been met the family will be consulted and a suitable time and day for infusion will be arranged. This will coincide with the availability of the staff at the hospital for support purposes. As part of the on-going clinical care the patient's pre medication regime and enzyme dose will be reviewed in clinic regularly to ensure it remains appropriate.

## 6 Results

Two patients had to return to the hospital setting due to difficulties with venous access at home, one returned home after replacement of a TIVAD and the other returned back to home infusions with continued cannulation after being referred for a TIVAD in the future. One further patient developed IRRs at home so returned to the hospital setting for two infusions whilst their pre-medication regime and rate increments were altered. This patient has now had no further IRRs within the homecare setting.

## 7 Discussion

The transition into homecare needs to be carefully managed and each patient must be considered on an individual basis. Enzyme replacement therapy for MPSIVA is an effective treatment for this cohort of patients. However due to the skeletal nature and the natural history of MPSIVA further long term outcome studies will be crucial in order to determine the long term effect of Elosulfase-alpha.

Commencing on ERT can be a significant burden on both the patient and their family especially when patients are required to travel a significant distance to the nearest infusion centre. Out of the fourteen patients that are currently on Elosulfase alpha in Manchester only three of these were from the local area (within a 5 mile radius). Transitioning to homecare can ease this burden significantly as families can plan the infusions around school or work commitments. In the authors experience patients often have some initial anxiety about transitioning into homecare however once transitioned they adjust very quickly due to increased independence as treatment can be integrated into normal activities of daily living ultimately improving quality of life. On average our patients would spend 4 h per week travelling for treatment and would miss 1 day per week of school, parents often reported that their child was often tired the following day after treatment due to travel and the effects of some pre-medications which meant then often had to leave school early or struggled to concentrate in class. In addition, home therapy can improve compliance and can decrease the pressure on resources at the specialist centre [15], [16]. All fourteen of our current patient cohort has demonstrated 100% compliance with home treatment. Once established with homecare, many children have their infusion administered in school which allows for them to attend school full time so that their education does not suffer as a result of treatment. Infusions will only be commenced in school if this felt to be appropriate and it is desired by the patient and their family.

Home treatment can also have a positive effect (in the UK) from a cost perspective; drugs that are dispensed from a community pharmacy are exempt from VAT (value added tax) as the current rate in the UK is 20%. Home treatment can be seen to benefit the health service as well as the family. The homecare teams also relay essential information to the hospital team by having regular direct contact with the family. This can be particularly effective when caring for families with complex social situations or safeguarding issues.

Conversely, homecare is not necessarily suitable for all patients. This may be due to various factors such as parental anxieties, severe airway disease, untenable behaviour, IRR's, or family and environmental difficulties. These issues can be discussed at clinic and management plans put in place to assist the family to transition into a homecare setting if this is deemed to be the best management of the individual patient.

## 8 Conclusion

In this paper, we describe the center's wealth of experience with MPS IV and homecare, based on this we demonstrate general criteria in assessing eligibility for home therapy. It is hoped that the criteria presented in this report will facilitate the safe transfer of MPS IVA patients into home therapy. As stated in Table 1, Table 2 even those with the most severe manifestations of MPS IVA or with significant IRR's can be managed safely at home providing all criteria are met prior to discharge. Home therapy is ultimately more convenient for the family in the context of a lifelong, weekly, IV therapy and allows for patients to have treatment without a significant negative impact upon their quality of life.

## Conflicts of interest

NF and SAJ have received honoraria and travel grants from BioMarin and Genzyme.

## Source of funding/Acknowledgements

None.

## References

[1] Neufield E.F., Muenzer J., Scriver C.R., Beaudet A.L., Sly W.S., Valle D. (2001). The mucopolysaccharidoses. The Metabolic and Molecular Bases of Inherited Disease.

[2] Montano A.M., Tomatsu S., gottesman G.S., Smoth M., Orii T. (2007). International Morquio A registry: clinical manifestation and natural course of Morquio A disease. J. Inherit. Metab. Dis..

[3] Harmatz P., Mengel K.E., Giugliani R., Valayannopolos V., Lin S.P., Parini R., Guffon N., Burton B.K., Hendriksz C.J., Mitchell J., Martins A., Jones S., Guelbert N., Vellodi A., Hollak C., Slasor P., Decker C. (2013). The Morquio A clinical assessment program: baseline results illustrating progressive multisystemic clinical impairments in Morquio A subject. Mol. Genet. Metab..

[4] Hendriksz C.J., Al-Jawad M., Berger K.I., Hawley S.M., Lawrence R., McArdle C., Summers C.G., Wright E., Braunlin E. (2013). Clinical overview and treatment options for non-skeletal manifestations of mucopolysaccharidosis type IVA. J. Inherit. Metab. Dis..

[5] Regier D.S., Oetgen M., Tanpaiboon P., Pagon R.A., Adam M.P., Ardinger H.H. (2016). Mucopolysaccharidosis type IVA. GeneReviews®. Seattle.

[6] Hendriksz C.J., Berger K.I., Giugliani R., Harmatz P., Kampmann C., Mackenzie W.G., Rainman J., Villarreal M.S., Savarirayan R. (2015). International guidelines for the management and treatment of Morquio A syndrome. Am. J. Med. Genet. Part A.

[7] Davison J.E., Kearney S., Horton J., Foster K., Peet A.C., Hendriksz C.J. (2013). Intellectual and neurological functioning in Morquio syndrome (MPS Iva). J. Inherit. Metab. Dis..

[8] Tomatsu S., Montano A.M., Nishioka T., Gutierres M.A., Pena O.M., Tranda firescu G.G., Lopez P., Yamaguchi S., Noguchi A., Orii T. (2005). Mutation and polymorphism spectrum of the GALNS gene in mucopolysaccharidosis IVA (Morquio A). Hum. Mutat..

[9] Biomarin (2014). Vimizim (Elosulfase Alfa) Product Monograph.

[10] Pharmaceuticals Biomarin (2013). Vimizim (Elosulfase alpha) for the Treatment of Mucopolysaccharidosis Type IVA (Morquio Syndrome): Briefing Document for the Endocrinologic and Metabolic Drugs Advisory Committee 2013. http://www.fda.gov/downloads/advisorycommittee/ucm375127.pdf.

[11] Food and Drug Administration Vimizim (Elosulfase alpha) injection, for intravenous use: US prescribing information; 2014. http://www.accessdata.fda.gov/drugsatfda_docs/label/2014/125460s000lbl.pdf.

[12] Hendriksz C.J., Burton B., Fleming T.R. (2014). Efficacy and safety of enzyme replacement therapy with BMN 110 for Morquio A Syndrome (mucopolysaccharidosis IVA): a phase 3 randomised placebo-controlled study. J. Inherit. Metab. Dis..

[13] Hughes D.A., Milligan A., Mehta A. (2007). Home therapy for lysosomal storage disorders. Br. J. Nurs..

[14] Milligan A., Hughes A., Goodwin S., Richfield L., Mehta A. (2006). Intravenous enzyme replacement therapy: better in home or hospital?. Br. J. Nurs..

[15] Bagewadi s Roberts J., Mercer J., Jones S., Stephenson J., Wraith J.E. (2008). Home treatment with Elaprase and Nagalzyme is safe in patients with mucopolysaccharidosis types II and VI, respectively. J. Inherit. Metab. Dis..

[16] Burton B.K., Weisman C., Paras A., Kim K., Katz R. (2009). Home infusion therapy is safe and enhances compliance in patients with mucopolysaccharidosis. Mol. Genet. Metab..

[17] Laraway S., Mercer J., Jameson B., Ashworth J., Hensman P., Jones S. (2016). Outcomes of long-term treatment with laronidase in patients with mucopolysaccharidosis type I. J. Pediatr. Nov..

[18] Brunelli MJ, Atallah AN, da Silva EMK (2016) Enzyme replacement therapy with galsulfase for mucopolysaccharidosis type VI. Cochrane Database Syst. Rev. 10.1002/14651858.CD009806.pub2 26943923

[19] Tomanin R., Zanetti A., D'Avanzo F., Rampazzo A., Gasparotto N., Parini R., Pascarella A., Concolino D., Procopio E., Fiumara A., Borgo A., Frigo A., Scarpa M. (2014). Clinical efficacy of enzyme replacement therapy in paediatric Hunter patients, an independent study of 3.5 years. Orphanet J. Rare Dis..

[20] Ceravolo F., Mascaro I., Sestito S., Pascale E., Lauricella A., Dizione E., Concolino D. (2013). Home treatment in paediatric patients with Hunter syndrome: the first Italian experience. Ital. J. Pediatr..

[21] Concolino D., Amico L., Cappellini M.D., Cassinerio E., Conti M., donate M.A., Favlo F., Fiumara A., Maccarone M., Manna R., Musumeci M.B., Nicoletti A., Nistico R., Papadia F., Parini R., Peluso D., Pensabene L., Pisanin A., Pistone G., Rigoldi M., Romani I., Tenuta M., Torti G., Veroux M., Zachara E. (2017). Home infusion program with enzymereplacement therapy for Fabry disease: the experience of a large Italian collaborative group. Mol. Genet. Metab. Rep..

